# Audiometric Characteristics and Tinnitus Features in a Cohort of 170 Spanish Patients

**DOI:** 10.3390/audiolres11040053

**Published:** 2021-11-03

**Authors:** María Cuesta, Pedro Cobo

**Affiliations:** Institute of Physical and Information Technologies (ITEFI), Spanish National Research Council (CSIC), Serrano 144, 28006 Madrid, Spain; m.cuesta@csic.es

**Keywords:** tinnitus, hearing loss, tinnitus handicap inventory

## Abstract

Background: Tinnitus is a rather prevalent, quite heterogeneous, and difficult to treat auditory disorder. The aim of this article is to provide the design and results of a cross-sectional study related to audiological and tinnitus features in a group of 170 Spanish patients. Methods: Audiometric characteristics were assessed on the basis of the pure-tone audiometry of both ears in 170 tinnitus patients and 85 control subjects. The audiometric status of each tinnitus participant was assessed on the basis of the average auditory threshold (*AAT*) in the whole frequency range (from 125 Hz to 8 kHz), and low (from 125 Hz to 2 kHz) and high (from 3 kHz to 8 kHz)-frequency intervals. Tinnitus features were evaluated through personal interview with patients and included tinnitus duration, laterality, pitch, sound, and distress (Tinnitus Handicap Inventory, *THI*). Correlational analysis was carried out between audiological (*AAT*) and tinnitus (*THI*) variables. Results: A very weak Spearman rank correlation factor is found between both variables. Conclusions: The subjective outcome of tinnitus distress (*THI*) was not correlated with the objective measure of hearing loss (*AAT*) in our cohort.

## 1. Introduction

Tinnitus, a phantom auditory perception in the absence of any sound source internal or external to the body [1,2], is a rather prevalent, quite heterogeneous, and difficult to treat auditory disorder. Although the figures of tinnitus prevalence are variable across studies, it is mostly accepted that 0.5–1% of the population in industrialized countries suffers from severe tinnitus [3], which produces a handicap or distress that can considerably deteriorate quality of life. In most patients, tinnitus is accompanied by sleep disturbance, annoyance, panic, stress, anxiety, or depression [4].

Tinnitus is known to be a heterogeneous disorder in several dimensions [5]: perception (laterality, pitch and type of sound), multiple factors of risk (different kinds of hearing loss, vestibular troubles, chronic headache, neck and temporomandibular disorder, psychiatric condition), related comorbidities (hyperacusis, attention problems, emotional stress), associated distress (psychological reactions to tinnitus), and large variability in the response to treatments. Heterogeneity contributes to the difficulty of treatment of tinnitus. Although there is no medicine specifically approved to treat tinnitus, there are many therapeutic approaches to alleviate it, including sound-based treatment [6,7], psychological intervention [8], electrical or magnetic stimulation [9], and combinations of some of the preceding modalities [10,11].

The relationship between tinnitus and hearing loss (HL) is an intriguing issue of crucial importance for research, as well as for clinical practice. According to Eggermont, the prevalence of tinnitus and the prevalence of HL higher than 25 dB are approximately related by a cubic-root dependence [12]. It has been proposed that tinnitus can arise as an aberrant plastic compensation of the neural part of the auditory system in response to some deafferentation from the peripheral part (outer, middle, or inner ears) through three mechanisms: hyperactivity (increment of the spontaneous activity), hypersynchrony, and reorganization of the tonotopic map [13]. The most usual cause of deafferentation to the auditory system is HL. Whilst HL is a principal risk factor to develop tinnitus, most people with HL do not suffer from tinnitus. Furthermore, between one-tenth and one-third of tinnitus patients have apparently normal hearing (HL less than 25 dB) [3,14], although this could be due to several reasons: (1) especially for high-pitched tinnitus, normal hearing at frequencies up to 8 kHz does not exclude cochlear deafferentation [15]; (2) hidden hearing losses (or cochlear synaptopathy) can occur, which are not detected by conventional audiometry [16]; (3) patients with apparent normal audiograms can show notched hearing losses when they are assessed with fine-step audiometry [17].

To gain insight into the relationship between audiological characteristics and tinnitus features, correlational studies in tinnitus subjects can be relevant. Therefore, the main aim of this article was to present such a correlational analysis in a cohort of 170 Spanish subjects.

## 2. Materials and Methods

### 2.1. Subjects

A total of 170 volunteers with tinnitus and 85 without tinnitus were recruited for this study, which was approved by the Bioethics Subcommittee of our Institution. Written informed consent was provided for all participants. Table 1 summarizes the mean and standard deviation (SD) age of the participants.

### 2.2. Audiometric Measurements

**Figure 1:**
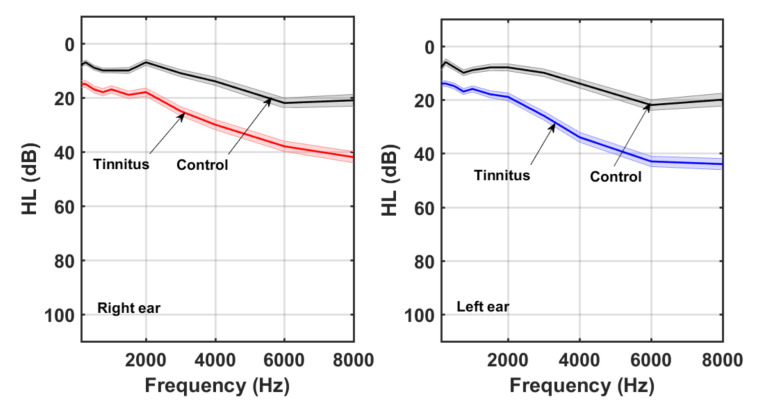
Right and left ear averaged HL curves for tinnitus and control participants.

Pure-tone thresholds of both ears were assessed for each participant, using a Clinic Audiometer GSI 60, at 11 frequencies (125, 250, 500, 750, 1000, 1500, 2000, 3000, 4000, 6000, and 8000 Hz). Figure 1 shows the average left and right HL curves for all participants. For each ear, the average HL curves for tinnitus and control were superimposed. The shaded area around each average HL curve represents the standard error (SE) ($\mathrm{SD}/\sqrt{N}$). As expected, the HL increased with frequency, with a greater slope in the case of tinnitus group. A slight bias toward greater HL at higher frequencies was observed in the left ear.

Average audiometric thresholds (*AAT*) were calculated for the left and right ears as

(1) $$AAT=\frac{1}{Nf}\overset{Nf}{\underset{1}{\sum }}HL\left(f_{i}\right),$$

where *HL(f_i_)* denotes the HL values at each frequency, and *Nf* is the number of frequencies. Additionally, low-frequency *AAT*, *AAT_LF_*, was defined as the average for the seven frequencies from 125 to 2000 Hz, and high-frequency *AAT*, *AAT_HF_*, was defined as the average for the four frequencies from 3000 to 8000 Hz.

*AAT*, *AAT_LF_*, and *AAT_HF_* were then used to define two hearing subgroups [18]:

1. Hearing impaired (HI) subgroup: subjects with any (*HL*(*f_i_*)) ≥ 40, *AAT* ≥ 30, or $[AAT_{HF}-AAT_{LF}]\geq 17;$
2. Normal hearing (NH) subgroup: all other subjects.

**Figure 2:**
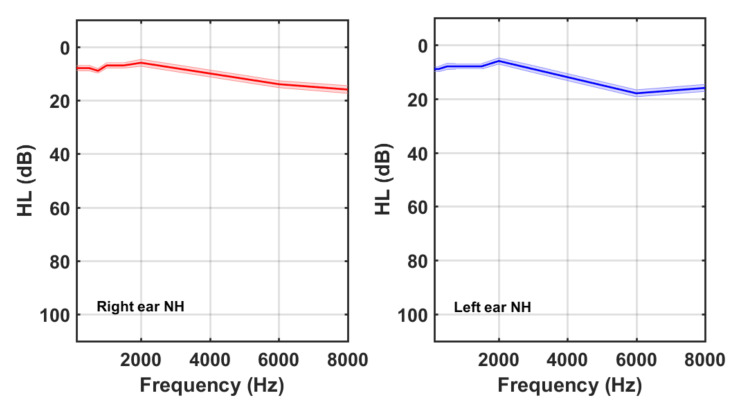
Right and left ear averaged HL curves for tinnitus participants of the NH subgroup.

**Figure 3:**
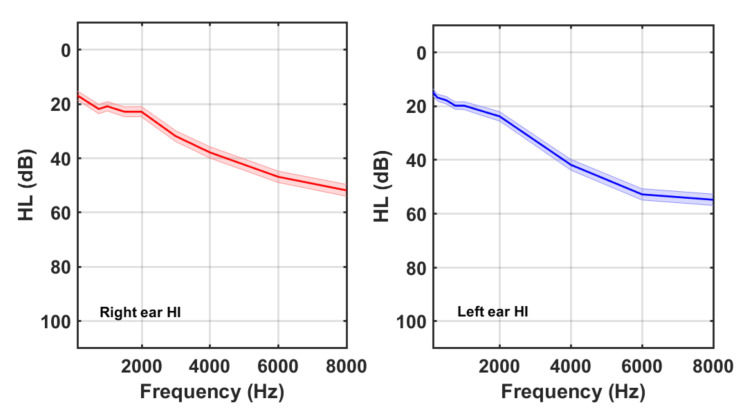
Right and left ear averaged HL curves for tinnitus participants of the HI subgroup.

The demographic characteristics of both groups are summarized in Table 2. It is noticeable that 29% (49/170) of the tinnitus participants had normal hearing. Figure 2 and Figure 3 show the average HL curves for the NH and HI subgroups, respectively. As expected, average HL for the NH subgroup remained lower than 20 dB. Participants of the HI subgroup, on the other hand, exhibited an abrupt fall at frequencies above 2 kHz. Again, larger HLs at higher frequencies were detected in the left ear of the HI subgroup.

### 2.3. Tinnitus Assessment

The responses of the tinnitus subjects to a clinical evaluation sheet were used to assess their tinnitus features, including the lateralization (left, right, or bilateral), duration (in months), and associated comorbidities of their tinnitus. Furthermore, anamnesis (clinic history, possible etiology, and previous tinnitus treatments), and tinnitus-related distress was reported through a version of the Tinnitus Handicap Inventory (*THI*) translated to and validated in Spanish [19].

A custom-designed graphical user interface (GUI) was used to evaluate the tinnitus pitch of participants. A bandpass noise controlled by two parameters, the central frequency and the −6 dB band around this frequency, is generated by this GUI. Therefore, the generated sound is a tone when the bandwidth is very narrow (<0.1%, for instance), a ringing for a bandwidth lesser than 10%, and a hissing for a wideband greater than 10%. A bracketing procedure was applied to find the type and pitch of the sound that most closely matched the tinnitus of the patient. Table 3 summarizes the tinnitus features of both tinnitus subgroups.

One of the questions responded by the patients in the evaluation sheet included the assignment of their tinnitus to the most probable cause. According to their responses, the predominant tinnitus etiology was emotional trouble (35%, stress, depression, anxiety, and obsessive–compulsive disorder), followed by HL (25%), overexposure to noise (15%), tube dysfunction (5%), ear surgery (4%), idiopathic (4%), and others (12%, head trauma, ototoxicity, otitis, rhinitis and sinusitis, barotrauma, cervical troubles, Meniere, and hydrocephaly).

## 3. Results

**Figure 4:**
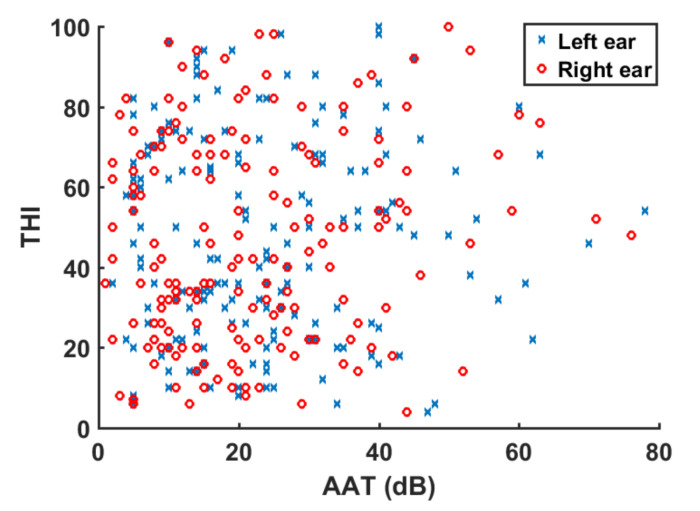
Scatter plot of *AAT* versus *THI* for the tinnitus subjects.

A correlational study was carried out between *AAT* and *THI* variables for the total tinnitus group and the NH and HI subgroups. To identify and test the strength of relationships between variables, Spearman rank correlation factors were calculated. Positive (negative) correlation factors between two variables denote that both increase (decrease) monotonically. Thus, very weak correlation was obtained for |*ρ*| ≤ 0.2, weak was obtained for 0.2 < |*ρ*| ≤ 0.4, moderate was obtained for 0.4 < |*ρ*| ≤ 0.6, strong was obtained for 0.6 < |*ρ*| ≤ 0.8, and very strong was obtained for |*ρ*| > 0.8 [20]. Table 4 summarizes the correlation factors between audiological (*AAT*) and tinnitus variables (*THI*). Correlation factors pointed to a very weak correlation between variables. The weak correlation between these variables is illustrated in Figure 4, which shows a scatter plot of *AAT* versus *THI* for the 170 tinnitus patients.

Previous studies proved a high correlation between tinnitus pitch (TP) and the frequency at which HL = 50 dB (F50) [18]. The subjects of the HI subgroup in this cohort had an average (mean, SD) TP of (5252, 2670) Hz whilst the F50 for the left and right ears (see Figure 3) was 5400 Hz and 7400 Hz, respectively. Therefore, a high correlation was also found in our cohort between the tinnitus pitch and the F50 at the left ear for the HI tinnitus subjects.

## 4. Discussion

Our categorization of HL considered hearing impaired (HI) and normal hearing (NH) subtypes. HI is the predominant tinnitus subgroup (71%), followed by NH (29%). Usually, it is assumed that tinnitus is associated with HL. Manche et al. [21], for instance, showed that 95.6% of tinnitus participants in a cohort of 3255 patients had some kind of HL (conductive, sensorineural, or mixed). Other works reported tinnitus concurrent with normal HL in about one-third [14] or one-tenth [3] of all cases. In the sample of Wallhäusser-Franke et al. [22], 25% of those with unilateral tinnitus and 20% with bilateral tinnitus did not have an overt HL. Furthermore, they found in their sample a high correlation between HL at 2 kHz and future tinnitus loudness. However, current evidence suggests at least two subtypes that can be differentiated within a hearing loss × distress matrix that should not be lumped together [23]. For the former (HI in the present study), tinnitus might be associated with HL or other inner-ear pathologies. Once the tinnitus sound reaches consciousness, it then merges or reacts with anxiety or distress. For the latter (NH in the present study), tinnitus might temporarily occur due to muscular or to cochlear pathologies undetected by the audiogram, or to a less well functioning efferent auditory system [22]. Nonetheless, once the stimulus is perceived, it is yet again reacted to with anxiety or distress, and the resulting vicious cycle contributes to the maintenance of the phenomenon. Furthermore, bear in mind that normal pure-tone audiometric thresholds do not exclude high-frequency or hidden hearing losses [15,16].

Concerning the tinnitus lateralization (Table 3), 49% of subjects perceived their tinnitus bilaterally, 35% perceived it in the left ear, and 16% perceived it in the right ear. Some studies also reported a bias toward the left ear [24,25], but some others found that subjects heard their tinnitus predominantly in the right ear [26]. We do not have a rational explanation for the bias of tinnitus sound toward the left ear in our cohort.

Regarding the tinnitus sound in our cohort (Table 3), tonal, hissing, and ringing sounds had prevalences of 34%, 36%, and 30%, respectively. These figures differ from those of the Tinnitus Archive of the Oregon Health State University (OHSU) [27], where tonal, ringing, and hissing are also the more recurrent tinnitus sounds, but with a distinct predominance.

The subjective outcome of tinnitus distress (*THI*) and the objective measure of hearing loss (*AAT*) were not correlated in our cohort. It appears that the stress reaction to neutral sound constitutes a psychological problem that is key to understand chronic tinnitus perception [28]. HL has been identified as the most relevant etiologic factor and as the probable trigger for tinnitus, although nonauditory factors may ameliorate or worsen the tinnitus [22].

Therefore, it seems that HL can be a risk factor for developing tinnitus, although the grade of tinnitus-related distress may be more determined by emotional aspects of the patient response. A similar result was found by other authors [29,30,31,32,33]. Some others, however, suggested that hearing deficit seems to increase the perceived distress of tinnitus [34] or even found a statistically significant correlation between the elevation of audiometric thresholds and the intensity of tinnitus as measured through a visual analog scale [35].

This work focused on the relationship between audiological and tinnitus-related distress. However, it is worth highlighting recent studies that investigated interactions between personality factors and subjectively experienced stressors in accounting for tinnitus-related distress [36]. They found that tinnitus-related distress was mediated by differential interactions between personality factors and perceived stress dimensions. Machine learning has been applied to reliably identify baseline features recorded prior to treatment commencement that characterize tinnitus-related distress after treatment [36]. The identification of key features can contribute to an improved understanding of multifactorial contributors to tinnitus-related distress and, thus, to multimodal treatment strategies. These studies suggest emotion-focused treatment strategies as a promising new direction for alleviating tinnitus distress [36,37].

It has been reported that chronic tinnitus in the absence of identifiable hearing pathology (HL subgroup in the present study) frequently overlaps with other somatoform disorders [38,39]. Some studies have found a substantial comorbidity between somatization and tinnitus, suggesting that there might be a spectrum of psychological factors having varying degrees of influence on tinnitus-related distress [38]. This is especially true for decompensated (highly distressing) tinnitus which is characterized by additional depressive, anxiety, or psychosomatic symptoms. Hiller et al. [38] found that 48% of patients with decompensated tinnitus also presented a somatoform disorder, whereas this was not the case for patients of the decompensated subgroup. In addition, Boecking et al. [40] found some gender differences in the health-related quality of life of chronic tinnitus patients regarding possible mutual influences of tinnitus-related distress and somatoform disorders. Specifically, their analyses revealed a central role of depressive symptoms for women and anxiety symptoms for men [40].

## 5. Conclusions

This paper reported the results of a cross-sectional study of audiometric and tinnitus characteristics in a sample of 170 Spanish tinnitus subjects. Participants were classified into two subgroups, considering their HL curves: hearing impaired (HI) and normal hearing (NH). The more prevalent subgroup was the HI (71%); 29% of the cohort, on the other hand, had apparent NH.

Tinnitus features were assessed through a personal interview to the tinnitus subjects and included laterality (left ear, right ear, or bilateral), type of sound (tonal, ringing, or hissing), duration, and initial *THI*. Tinnitus pitch was evaluated by matching the bandpass noise, generated by a custom-designed GUI, closely to the tinnitus sound. The findings are summarized below.

1. In this cohort, 35% of subjects allocated their tinnitus to the left ear, and 16% assigned their tinnitus to the right ear, whilst, in 49% of subjects, the tinnitus was bilateral.
2. The most frequent tinnitus sound was hissing (36%), followed by tonal (34%) and ringing (30%).
3. The predominant tinnitus etiology (35%) was emotional troubles (stress, depression, anxiety, and obsessive–compulsive disorder), followed by HL (25%), overexposure to noise (15%), tube dysfunction (5%), ear surgery (4%), idiopathic (4%), and others (12%, including head trauma, ototoxicity, otitis, rhinitis and sinusitis, barotrauma, cervical troubles, Meniere, and hydrocephaly).
4. The subjective outcome of tinnitus distress (*THI*) was not correlated with the objective measure of hearing loss (*AAT*), whether for the HI or NH subgroups. Therefore, it seems that hearing loss can become a risk factor for triggering tinnitus, although the grade of tinnitus-related distress may be more determined by emotional aspects of the patient response.

## Figures and Tables

**Table 1:** Age description of participants.

	Tinnitus Group			Control Group		
	Total	Males	Females	Total	Males	Females
*N*	170	110	60	85	38	47
Age (mean)	50	51	49	44	47	43
Age (SD)	11	11	10	13	13	12

**Table 2:** Demographic characteristics of subjects in NH and HI subgroups.

	NH Subgroup	HI Subgroup
Total	49 (29%)	121 (71%)
Males	26 (53%)	84 (69%)
Females	23 (47%)	37 (31%)

**Table 3:** Tinnitus features of patients in NH and HI subgroups.

		Total	NH Subgroup	HI Subgroup
Duration, in months (mean, SD)		75, 102	66, 110	79, 99
Pitch, in Hz (mean, SD)		5200, 3016	5085, 3764	5262, 2670
THI, % (mean, SD)		47, 25	45, 26	47, 25
Lateralization	Bilateral, *N* (%)	84 (49%)	26 (53%)	59 (49%)
	Left ear, *N* (%)	59 (35%)	17 (35%)	42 (35%)
	Right ear, *N* (%)	27 (16%)	6 (12%)	20 (16%)
Tinnitus sound	Hissing, *N* (%)	62 (36%)	16 (33%)	47 (39%)
	Ringing, *N* (%)	50 (30%)	16 (33%)	34 (28%)
	Tonal, *N* (%)	58 (34%)	17 (34%)	40 (33%)

**Table 4:** Spearman rank correlation factors between variables.

	*AAT* (All)		*AAT* (NH)		*AAT* (HI)	
	Left Ear	Right Ear	Left Ear	Right Ear	Left Ear	Right Ear
*THI* (All)	0.06	0.094				
*THI* (NH)			−0.078	−0.06		
*THI* (HI)					0.057	0.10

## Data Availability

The data are not publicly available due to the confidentiality clause of the informed consent form.
